# Comparing conformal, arc radiotherapy and helical tomotherapy in craniospinal irradiation planning

**DOI:** 10.1120/jacmp.v15i5.4724

**Published:** 2014-09-08

**Authors:** Pamela A. Myers, Panayiotis Mavroidis, Nikos Papanikolaou, Sotirios Stathakis

**Affiliations:** ^1^ Department of Radiation Oncology Baylor College of Medicine Houston TX USA; ^2^ Department of Radiation Oncology University of Texas Health Science Center at San Antonio San Antonio TX USA

**Keywords:** radiation biology, pediatric, craniospinal axis irradiation, biologically effective uniform dose, complication‐free tumor control probability

## Abstract

Currently, radiotherapy treatment plan acceptance is based primarily on dosimetric performance measures. However, use of radiobiological analysis to assess benefit in terms of tumor control and harm in terms of injury to normal tissues can be advantageous. For pediatric craniospinal axis irradiation (CSI) patients, in particular, knowing the technique that will optimize the probabilities of benefit versus injury can lead to better long‐term outcomes. Twenty‐four CSI pediatric patients (median age 10) were retrospectively planned with three techniques: three‐dimensional conformal radiation therapy (3D CRT), volumetric‐modulated arc therapy (VMAT), and helical tomotherapy (HT). VMAT plans consisted of one superior and one inferior full arc, and tomotherapy plans were created using a 5.02 cm field width and helical pitch of 0.287. Each plan was normalized to 95% of target volume (whole brain and spinal cord) receiving prescription dose 23.4 Gy in 13 fractions. Using an in‐house MATLAB code and DVH data from each plan, the three techniques were evaluated based on biologically effective uniform dose ($\bar{\bar{D}}$), the complication‐free tumor control probability ($P_{+}$), and the width of the therapeutically beneficial range. Overall, 3D CRT and VMAT plans had similar values of $\bar{\bar{D}}$ (24.1 and 24.2 Gy), while HT had a $\bar{\bar{D}}$ slightly lower (23.6 Gy). The average values of the $P_{+}$ index were 64.6, 67.4, and 56.6% for 3D CRT, VMAT, and HT plans, respectively, with the VMAT plans having a statistically significant increase in $P_{+}$. Optimal values of $\bar{\bar{D}}$ were 28.4, 33.0, and 31.9 Gy for 3D CRT, VMAT, and HT plans, respectively. Although $P_{+}$ values that correspond to the initial dose prescription were lower for HT, after optimizing the $\bar{\bar{D}}$ prescription level, the optimal $P_{+}$ became 94.1, 99.5, and 99.6% for 3D CRT, VMAT, and HT, respectively, with the VMAT and HT plans having statistically significant increases in $P_{+}$. If the optimal dose level is prescribed using a radiobiological evaluation method, as opposed to a purely dosimetric one, the two IMRT techniques, VMAT and HT, will yield largest overall benefit to CSI patients by maximizing tumor control and limiting normal tissue injury. Using VMAT or HT may provide these pediatric patients with better long‐term outcomes after radiotherapy.

PACS number: 87.55.dk

## I. INTRODUCTION

Currently, treatment plan acceptance is largely based on dosimetric data such as dose‐volume histogram (DVH) statistics for targets, and normal tissues and isodose line distributions. While these parameters do offer insight into the dosimetric capability of a given plan, the radiobiological effects on the involved tumor and normal tissues may convey a significantly different outlook regarding the acceptability of the plan. When comparing two different plans, which for example may differ in the treatment modality used (e.g., intensity‐modulated radiation therapy (IMRT) versus three‐dimensional conformal radiation therapy (3D CRT)), their dosimetric values may be similar, but using radiobiological analysis the plans may show significant differences. The biologically effective uniform dose ($\bar{\bar{D}}$) is defined as the dose which, when delivered uniformly, will result in the same probability of tumor control or normal tissue complications as the actual inhomogeneous dose distribution delivered to the patient.^(1)^ Using the concepts of the $\bar{\bar{D}}$ and complication‐free tumor control probability ($P_{+}$), the quality of a given plan in terms of treatment outcome of the target and surrounding critical organs can be assessed.^(2)^

The knowledge that can be gained from a radiobiological assessment of a treatment plan may be extremely beneficial regarding the outcome of the treatment, as well as the quality of life for the patient after radiation therapy. Pediatric craniospinal axis irradiation (CSI) patients may be some of the best candidates for this kind of analysis for several reasons. Younger patients are known to be more sensitive to radiation than adults due to their ongoing development and continued rapid cell proliferation as compared to a fully‐matured individual.^(3)^ Also, due to their early age of treatment, their life expectancies and quality of life have to be seriously considered when deciding on the best course of treatment. CSI is used to treat central nervous system (CNS) tumors and includes a target volume consisting of the entire brain and the spinal column. More specifically, in children treated with CSI, there is a large percentage of the patient's anatomy that is exposed to some level of radiation dose due to the large target involved in the treatment.^4^, ^5^ For these reasons, a treatment plan evaluation, which would use the patient‐specific planned dose distribution and would focus on normal tissue complication rates and tumor control probabilities, should be performed.

As a consequence of recent technological advances, it is now possible to treat these pediatric CSI patients, as well as the majority of the patients treated with radiotherapy, with different types of treatment modalities. Historically, CSI has been treated using 3D CRT consisting of opposing whole brain and posterior spinal fields. With the increased use of IMRT techniques in the clinic today, these patients can also be treated with multileaf collimator (MLC)‐based modalities such as step‐and‐shoot IMRT, sliding window IMRT, volumetric‐modulated arc therapy (VMAT), and helical tomotherapy (HT). IMRT techniques have shown to increase the prescription dose to the target and achieve a better sparing of the surrounding critical tissues compared to the traditional 3D CRT. However, the dosimetric information alone does not always point out the radiobiologically superior treatment for the patient. The outcome of the plan can depend heavily on not only the dose given to normal tissues and the target, but also the specific dose distribution and the dose response of the involved organs. Relevant studies have been published concerning these outcomes for adult patients of varying disease sites for different treatment modalities using dosimetric and radiobiological analyses.^6^, ^7^ These studies, however, are limited in their applicability to pediatric CSI patients due to the different radiosensitivity that characterizes these young patients.

This study aims to compare three treatment modalities that are commonly used for pediatric CSI treatment delivery based on dosimetric and radiobiological measures such as the $\bar{\bar{D}}$ and $P_{+}$ indices. The modalities that are examined are the three‐dimensional conformal radiation therapy, the SmartArc VMAT, and the helical tomotherapy. Using the treatment information of the plans that were generated with each of these techniques for a population of pediatric patients (n=24), the most radiobiologically effective treatment option in terms of maximizing tumor control while limiting normal tissue complication probabilities is determined.

## II. MATERIALS AND METHODS

### A. Treatment planning techniques

A total of twenty‐four (n=24) pediatric patients, including 11 females and 13 males, (median age: 10 years, range: 2‐18 years), who were treated for various central nervous system (CNS) diseases (14 medulloblastoma, 3 germ cell tumors, 2 primitive neuroectodermal tumors (PNET), 2 pre‐B‐cell acute lymphoblastic leukemia (ALL), 1 meningeal carcinomatosis, 1 pineoblastoma, and 1 atypical teratoid rhabdoid tumor of the brain) with CSI were retrospectively planned with three‐dimensional conformal radiation therapy (3D CRT), and two IMRT techniques: volumetric‐modulated arc therapy (VMAT) and helical tomotherapy (HT). In this study, pediatric patients were considered those patients who were less than 20 years of age at the time of treatment. For each of the three plans, for each patient a total dose of 23.4 Gy (13 fractions of 1.8 Gy) were prescribed (even though different prescription doses are used in different tumor cases, in this study the same prescription was used to facilitate the intercomparison across all the examined patient cases). The plan approval clinical criteria required that 95% of the planning target volume (PTV) should receive at least the prescription dose, and the dose to the involved critical organs (lungs, heart, kidneys, orbits, liver, colon, thyroid, and breasts for the female patients) should be below their tolerance limits as defined in the QUANTEC report.^(8)^ The PTV encompassed the whole brain and the entire spinal cord with a 0.7 cm isotropic expansion.^(7)^ The patient's respective physician delineated all the structures and defined the dose prescription prior to planning, and those factors were kept unchanged during the production of all the plans in each individual case. All the structures were delineated by the physician in the Pinnacle^3^ (Philips Medical, Fitchburg, WI) treatment planning system (TPS). Subsequently, the structures and the CT images were exported using the DICOM‐RT protocol to the TomoTherapy Hi·Art (TomoTherapy Inc., Madison, WI) TPS to ensure consistency among the three radiation modalities.

#### A.1 3D conformal radiation therapy (3D CRT)

**Figure 1:**
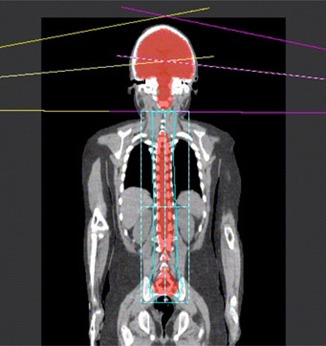
Illustration of the beam arrangement used in the 3D CRT radiation modality. In this technique, two lateral brain fields and one posterior spinal field are applied.

The Pinnacle^3^ TPS was used for creating the 3D CRT plans. Two opposing lateral fields were used to treat the whole brain, and one posterior field was used to treat the spinal cord. Depending on the size and anatomy of the patient, 6 MV, 18 MV, or a combination of these two beam energies was used in order to achieve the best possible coverage of the PTV with the prescription dose. More specifically, these plans were focused on maintaining a dose of at least 95% of the prescription and no more than 107% of the prescribed dose to the target volume. Facial blocking was used in the whole brain fields to protect structures in the jaw and mouth of the patient. Similarly, blocks surrounding the PTV were drawn for the spinal cord field to protect areas outside the target volume. The plans were created for a Varian 21EX linear accelerator (Varian Medical Systems, Palo Alto, CA) equipped with a 120 Millennium multileaf collimator (MLC). Gantry rotations were used to prevent divergence of the lateral brain fields into the orbits of the patient. The “gap match” method was used at the junction of the brain and spinal fields, as described by Khan.^(9)^ Figure 1 illustrates the three‐field setup of the 3D CRT treatment modality.

#### A.2 Volumetric‐modulated arc therapy (VMAT)

**Figure 2:**
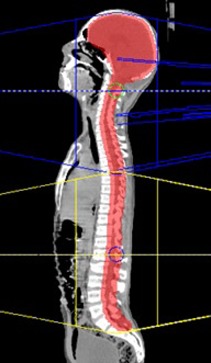
Illustration of the beam setup used in the SmartArc radiation modality. In this technique, one superior and one inferior treatment arc are applied.

The Pinnacle^3^ TPS was also used to plan the SmartArc VMAT cases. Similar to the 3D CRT plans, the dosimetric data of a Varian 21EX linear accelerator was used for planning along with a Millennium 120 leaf MLC. Two isocenters, one superior and one inferior, were located along the PTV of the patient, according to the length of the target, in order to encompass the entire PTV in one superior and one inferior beam arc, as shown in Fig. 2. Each full arc spanned from 1 to 359 degrees around the patient with a 4 degree gantry spacing. Optimization was performed on the plans until the previously mentioned plan criteria (95% of the PTV volume should receive at least the prescribed dose while the dose to the surrounding normal tissues should be kept below their individual tolerance limits) was achieved.

#### A.3 Helical tomotherapy (HT)

The TomoTherapy Hi·Art TPS was used to plan the helical tomotherapy treatments. The input parameters used prior to optimization were the dose criterion of 95% of the target volume receiving at least 23.4 Gy, 5.02 cm field width, pitch of 0.287, and a modulation factor of 2.0. The optimization of the plan was then performed similarly to that of the VMAT plans in order to satisfy the PTV coverage while delivering the lowest possible dose to the surrounding organs at risk.

### B. Radiobiological evaluation

In order to evaluate and compare the different treatment modalities, a number of radiobiological measures were employed. The Poisson model describes the dose‐response relationship of normal tissues and tumors according to the following expression:^2^, ^6^, ^10^, ^11^

(1) $$P(D)=\text{exp}\left(-e^{e\gamma -\alpha nd-\beta nd^{2}}\right)\approx \text{exp}\left(-e^{e\gamma -({\text{EQD}}_{2\text{Gy}}/D_{50})\cdot (e\gamma -\text{ln}\;\text{ln}2)}\right)$$

where *P(D)* is the probability of inducing tumor control or normal tissue complications when the tissue is irradiated with a uniform dose of ${EQD}_{2Gy}$, γ is the maximum normalized gradient of the dose response curve, and $D_{50}$ is the dose causing a 50% response. $EQD_{2Gy}$ is the 2 Gy equivalent dose and it is calculated by the following equation:

(2) $${\text{EQD}}_{2\text{Gy}}=D\cdot \left(\frac{1+\frac{d}{\alpha /\beta }}{1+\frac{2}{\alpha /\beta }}\right)=\frac{\text{BED}}{1+\frac{2}{\alpha /\beta }}$$

where 2 Gy represents the reference dose level used for the determination of the dose‐response parameters, *D* is the total physical dose, *d* is the dose per fraction and α*/*β is the organ‐specific dose that accounts for the fractionation characteristics of the tissue and at which the linear and quadratic components of cell killing are equal.^(3^) BED is the biological effective dose, which is a measure of the biological dose delivered to a tumor or organ and it is the theoretical total dose that would be required to produce a particular isoeffect using an infinitely large number of infinitesimally small dose fractions. BED has been used for comparing different treatment strategies.^12^, ^13^, ^14^ The relation of the parameters $D_{50}$ and γ with α and β that leads to equality of Eq. (1) is the following:

(3) $$\alpha =\frac{e\gamma -\text{ln}(\text{ln}2)}{D_{50}\left(1+\frac{d}{\alpha /\beta }\right)}\;\;\text{and}\;\;\beta =\frac{e\gamma -\text{ln}(\text{ln}2)}{D_{50}\left(\frac{\alpha }{\beta }+d\right)}$$

where $d_{ref}$ is the reference dose per fraction to which the parameter $D_{50}$ and γ refer.

The response of a normal tissue to a nonuniform dose distribution is calculated using the relative seriality model in which the volume effect is taken into account. The mathematical expression of the relative seriality model is given by the following equation:^(2,611^)

(4) $$P_{I}=1-\prod_{j=1}^{N_{organs}}\;(1-{\left[1-\prod_{i=1}^{M_{j}}{\left(1-P^{j}{\left(D_{i}\right)}^{S_{j}}\right)}^{\Delta v_{i}}\right]}^{\frac{1}{S_{j}}}$$

where $N_{organs}$ is the total number of organs at risk; $P^{j}(D_{i})$ as described by Eq. (1) is the probability of response of organ *j*, which is irradiated with dose $D_{i}$ and has the reference volume; $\Delta v_{i}$ is the fractional subvolume of the organ being irradiated compared to that of the reference volume; and $M_{j}$ is the total number of subvolumes in the organ. $S_{j}$ is the relative seriality parameter which describes the internal structure of the examined organ, and it is approximately unity for a completely serial structure and zero for a completely parallel structure.^(6^) For the estimation of the expected normal tissue complications, other models have also been proposed, such as the Lyman‐Kutcher‐Burman (LKB) model, the Parallel model, and the Critical Volume model.^15^, ^16^, ^17^, ^18^ Even though the LKB model is well known, the Relative Seriality model was applied in this study due to its previous extensive use in studies involving radiobiological evaluation or optimization of treatment techniques and due to the previous experience of the authors with this model and the relevant dose‐response parameters in the calculation of the normal tissue complication probability (NTCP) values.

Due to the fact that tumor control is achieved when the separate tumor cells are completely eradicated, it is assumed that tumors have a parallel structure. Consequently, the probably of benefit or tumor control is given by the following equation:

(5) $$P_{B}=\prod_{j=1}^{N_{tumors}}\left(\prod_{i=1}^{M_{j}}P^{j}{\left(D_{i}\right)}^{\Delta v_{i}}\right)$$

where $N_{tumors}$ is the total number of targets that are being treated.^(6^)

The dose‐response parameters used in this study are summarized in Table 1. These values were obtained from published data, which were based on adult patients.^6^, ^10^, ^11^, ^12^, ^13^, ^14^, ^15^, ^16^, ^17^, ^18^, ^19^, ^20^, ^21^, ^22^, ^23^, ^24^, ^25^, ^26^, ^27^, ^28^, ^29^ The uncertainties associated with these parameters are mostly of the order of 5% for $D_{50}$, 30% for γ, and 90% for s, which define the confidence interval of the entire dose‐response curve around its best estimate. These values were modified to correspond to the increased radiosensitivity of the pediatric patients by applying an effective radiation sensitivity factor of 3. This was done by using the $D_{50}$ and γ values of adults to calculate the effective radiosensitivities of the different tissues, which were then rescaled by the factor stated above. Subsequently, using the age‐adjusted effective radiosensitivities, the age‐adjusted values of $D_{50}$ and γ were calculated for each tissue (Table 1). This increase in sensitivity has been reported as the overall lifetime risk increase experienced by children versus adults.^(3)^

The two main radiobiological measures that are used to evaluate and compare the different treatment plans are the biologically effective uniform dose ($\bar{\bar{D}}$) and probability of controlling the tumor without causing severe injury to the normal tissues ($P_{+}$). The $P_{+}$ can be calculated by using (4), (5) according to the following expression:^(6)^

(6) $$P_{+}=P_{B}-P_{B\cap I}\approx P_{B}-P_{I}$$

$\bar{\bar{D}}$ is calculated using the following equality and subsequent equation:^(6)^

(7) $$P\left(\bar{\bar{D}}\right)=P(\vec{D})=>\bar{\bar{D}}=\frac{e\gamma -\text{ln}(-\text{ln}(P(\vec{D})))}{e\gamma -\text{ln}(\text{ln}2)}$$

where $\vec{D}$ is the three‐dimensional dose distribution. The $\bar{\bar{D}}$ is described as the uniform dose that would cause the same tumor control (or normal tissue complication, respectively) as the actual nonuniform dose distribution that is delivered to the patient.^(1)^ The $\bar{\bar{D}}$ is averaged over both the applied dose distribution and the dose‐response relations of the involved tissues. By plotting the response probabilities of the different tissues (PTV and OARs) against $\bar{\bar{D}}$, different treatment plans can be compared.^(6)^ In these plots, based on the inherent features of the $\bar{\bar{D}}$ concept, the position of the dose response curves of the PTV of the different plans are forced to coincide. So, the position of the dose response curves of the OARs will more clearly indicate which of the plans is superior based on an OAR comparison for the same PTV dose response.

**Table 1:** Summary of dose‐response parameters used in this study

*Tissue*	$D_{50}(Gy)$	*γ*	*s*	α/β	*Endpoint*
PTV	22.86	4.38	–	10.0	Control
Lung	17.20	1.70	0.01	3.0	Radiation pneumonitis
Kidney	16.11	4.55	0.004	3.0	Clinical nephritis
Eye	37.14	3.15	1.0	3.0	Blindness
Heart	40.40	1.68	1.0	3.0	Cardiac mortality
Liver	22.40	4.55	0.17	3.0	Liver failure
Thyroid	51.43	3.50	0.10	3.0	Radiation induced hyperthyroidism
Colon	31.60	5.43	0.69	3.0	Obstruction perforation/ulceration/fistula
Breast^a^	37.14	4.83	1.0	3.0	Necrosis

a For female patients only.

Another radiobiological measure that was used to compare the dose distributions of the three radiation modalities is the biological effective dose (BED).^30^, ^31^ The BED values of each target and OAR for each patient and modality were calculated from their corresponding DVHs based on the following expression:

(8) BED=∑i=1Ndi(1+di/nαβ)*Vi∑i=1NVi

where $d_{i}$ and $V_{i}$ are the dose and volume in each bin (i) of the DVH, *n* is the number of fractions of the treatment; whereas α and β are tissue‐specific parameters related to cell radiosensitivity, expressed in units of ${Gy}^{-1}$ and ${Gy}^{-2}$, respectively.

#### B.1 Assessment of radiobiological measures

Once treatment planning was complete for each patient and each modality, the dose‐volume histograms (DVH) were exported from the respective TPS for each organ used in this study (namely: PTV, lungs, kidneys, orbits, heart, liver, thyroid, colon, and breasts for female patients only). Using an in‐house MATLAB (The MathWorks Inc., Natick, MA) program designed to radiobiologically evaluate different treatment plans or dose distributions, the traditional 3D CRT and both the VMAT and HT plans were be compared.^(32)^ This program uses the previously discussed (2), (3), (4), (5), (6) to calculate the respective radiobiological quantities for each plan. At the same time, the mean dose to each of the organs at risk (OAR) and PTV is calculated using the corresponding DVH data. Along with the calculation of each of these quantities, the MATLAB code generates plots containing a schematic representation of the dosimetric and radiobiological results of the methods being compared. Using these plots, the code calculates the optimal $\bar{\bar{D}}$ that corresponds to the maximum $P_{+}$ value for each individual patient and treatment modality indicating at the same time the therapeutically beneficial range of each plan.

### C. Treatment plan comparison

The data from the MATLAB comparisons (3D CRT vs. VMAT vs. HT) was generated and recorded in order to perform an overall comparison between the different modalities. Average values over all the 24 patients were computed for the quantities $\bar{\bar{D}}$, $P_{+}$, $P_{B}$, and $P_{I}$ for both the prescribed dose and the optimal $\bar{\bar{D}}$. The most important factor in the comparison is the $P_{+}$ index since it provides the overall probability of complication‐free tumor control for each of the examined treatment modalities. This value expresses the overall benefit of the given plan in terms of treatment outcome. Comparisons between the averages of the three modalities were then performed as a means of expressing which modality would be most beneficial for the treatment of the examined cancer type. In all the comparisons, a paired *t*‐test was applied in order to investigate the statistical significant of the observed differences.

## III. RESULTS

### A. Radiobiological evaluation of examined treatment modalities

**Figure 3:**
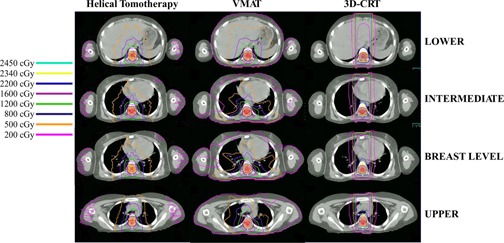
Illustration of the dosimetric axial distributions of the examined treatment modalities: helical tomotherapy, VMAT, and 3D CRT for a representative patient. For each modality, four slices at different anatomical levels are shown.

A representative patient (female; age 7; medulloblastoma) example for a modality comparison of axial dosimetric distributions is provided for reference in Fig. 3. 2, 3, 4 display the dosimetric (mean, maximum, and minimum dose) and radiobiological parameter values ($\bar{\bar{D}}$, BED and ${\text{EQD}}_{2\text{Gy}}$) for the three respective treatment modalities (3D CRT, VMAT, and HT) as an average over all 24 patients. The tumor control probability, $P_{B}$, for the PTV was 65.2% ± 1.4% for 3D CRT, 67.4% ± 2.8% for VMAT, and 56.6% ± 1.4% for HT. Respectively, the tissue injury probability, $P_{I}$, for all the other OARs was zero (VMAT and HT) or close to zero (0.00% ± 0.02% in right lung, 0.03% ± 0.16% in left kidney, and 0.5% ± 2.6% in right kidney) for 3D CRT. The minimum dose to the PTV is reported, while the maximum dose for each of the OARs is displayed. As can be seen from the tables, the injury probabilities for all techniques are very low. With the exception of the kidneys for the 3D CRT technique, injury probabilities are zero. At this level of dose prescribed, the results indicate that the risk of injuring the surrounding OARs is low for all treatment techniques. Knowing this, the dose level could be optimized to a higher value to maximize tumor control while still maintaining very low risks to the normal tissues.

**Table 2:** Summary of the dosimetric and radiobiological parameter values, which were calculated over all the patients, for the 3D CRT treatment modality. The minimum dose is reported for the PTV, whereas the maximum doses are reported for all other organs

*Organ*	$P_{B}/P_{I}$	$\bar{\bar{D}}$ *(Gy)*	$D_{mean}$ *(Gy)*	*BED (Gy)*	$EQD_{2Gy}$ *(Gy)*	$D_{max}/D_{min}$ *(Gy)*
PTV	65.2±1.4	24.1±0.1	24.2±0.1	26.1±0.9	21.5±0.7	21.7±0.7
Right lung	0.00±0.02	7.5±2.1	5.8±1.9	6.8±2.3	4.1±1.4	23.7±1.2
Left Lung	0.0±0.0	5.7±1.9	4.1±1.6	4.8±1.9	2.9±1.2	23.5±1.4
Right Kidney	0.5±2.6	12.3±1.7	5.3±2.9	6.1±3.6	3.6±2.1	22.3±2.2
Left Kidney	0.03±0.16	11.8±1.3	4.2±2.2	4.8±2.7	2.9±1.6	22.3±1.3
Right Eye	0.0±0.0	23.9±0.6	17.6±5.3	21.3±6.9	12.8±4.1	24.1±2.0
Left Eye	0.0±0.0	23.9±0.6	17.7±5.1	21.4±6.7	12.9±4.0	24.3±1.0
Heart	0.0±0.0	19.4±0.6	13.7±2.3	16.1±2.9	9.6±1.7	21.7±0.8
Liver	0.0±0.0	18.4±0.4	5.6±1.4	6.5±1.7	3.9±1.0	21.6±1.0
Thyroid	0.0±0.0	20.9±0.5	19.0±3.6	22.7±4.7	13.6±2.8	21.2±0.8
Colon	0.0±0.0	23.6±0.4	7.6±2.8	8.8±3.3	5.3±2.0	20.9±2.4
Right Breast	0.0±0.0	26.2±0.0	0.7±0.3	0.7±0.3	0.4±0.2	4.3±4.6
Left Breast	0.0±0.0	26.2±0.0	0.5±0.2	0.5±0.3	0.3±0.2	3.5±4.6

**Table 3:** Summary of the dosimetric and radiobiological parameter values, which were calculated over all the patients, for the volumetric‐modulated arc therapy (VMAT) treatment modality. The minimum dose is reported for the PTV, whereas the maximum doses are reported for all other organs

*Organ*	$P_{B}/P_{I}$ *(%)*	$\bar{\bar{D}}$ *(Gy)*	$D_{mean}$ *(Gy)*	*BED (Gy)*	$EQD_{2Gy}$ *(Gy)*	$D_{max}/D_{min}$ *(Gy)*
PTV	67.4±2.8	24.3±0.2	24.3±0.2	26.1±0.3	21.7±0.2	21.7±0.5
Right lung	0.0±0.0	6.7±0.9	6.4±0.9	6.9±1.2	4.1±0.7	18.1±1.9
Left Lung	0.0±0.0	6.7±0.9	6.4±0.9	6.9±1.0	4.2±0.6	18.1±2.1
Right Kidney	0.0±0.0	9.8±0.1	5.2±0.6	5.5±0.7	3.3±0.4	12.0±1.8
Left Kidney	0.0±0.0	9.8±0.0	5.1±0.6	5.4±0.7	3.3±0.4	11.8±1.5
Right Eye	0.0±0.0	22.1±0.3	15.2±1.3	17.6±1.7	10.6±1.0	21.5±1.3
Left Eye	0.0±0.0	22.1±0.2	14.9±1.3	17.2±1.7	10.4±1.0	21.3±1.2
Heart	0.0±0.0	10.4±0.3	4.4±0.7	5.2±2.5	3.1±1.5	9.8±1.7
Liver	0.0±0.0	14.9±0.0	4.5±0.6	4.8±0.6	2.9±0.4	10.7±1.3
Thyroid	0.0±0.0	20.5±0.0	7.8±0.8	8.5±1.0	5.1±0.6	12.8±1.4
Colon	0.0±0.0	23.6±0.4	5.1±0.9	5.4±1.0	3.2±0.6	11.0±3.8
Right Breast	0.0±0.0	26.2±0.0	1.6±0.2	1.6±0.3	0.9±0.2	2.4±0.7
Left Breast	0.0±0.0	26.2±0.0	1.5±0.2	1.5±0.2	0.9±0.1	2.4±0.6

**Table 4:** Summary of the dosimetric and radiobiological parameter values, which were calculated over all the patients, for the helical tomotherapy (HT) treatment modality. The minimum dose is reported for the PTV, whereas the maximum doses are reported for all other organs

*Organ*	$P_{B}/P_{I}$ *(%)*	$\bar{\bar{D}}$ *(Gy)*	$D_{mean}$ *(Gy)*	*BED (Gy)*	$EQD_{2Gy}$ *(Gy)*	$D_{max}/D_{min}$ *(Gy)*
PTV	56.6±1.4	23.7±0.1	23.6±0.1	25.3±0.1	21.1±0.1	22.6±0.4
Right lung	0.0±0.0	6.0±0.8	5.6±0.9	6.1±1.0	3.7±0.6	19.7±3.6
Left Lung	0.0±0.0	5.7±1.0	5.3±1.0	5.8±1.1	3.5±0.7	18.5±4.4
Right Kidney	0.0±0.0	9.9±0.3	5.2±1.0	5.6±1.2	3.4±0.7	14.3±4.2
Left Kidney	0.0±0.0	10.0±0.3	5.3±1.2	5.6±1.4	3.4±0.8	14.7±4.1
Right Eye	0.0±0.0	22.3±0.4	15.0±1.6	17.4±2.2	10.4±1.3	22.1±1.6
Left Eye	0.0±0.0	22.4±0.5	15.0±1.7	17.4±2.3	10.4±1.4	22.2±1.9
Heart	0.0±0.0	10.7±0.8	4.7±0.7	5.0±0.8	3.0±0.5	11.3±1.9
Liver	0.0±0.0	14.9±0.0	4.4±0.4	4.7±0.5	2.8±0.3	11.3±1.5
Thyroid	0.0±0.0	20.5±0.0	7.5±1.2	8.1±1.4	4.9±0.9	13.7±2.7
Colon	0.0±0.0	23.5±0.0	5.1±0.9	5.4±1.0	3.2±0.6	12.2±4.2
Right Breast	0.0±0.0	26.2±0.0	2.8±0.4	2.9±0.5	1.7±0.3	3.8±0.9
Left Breast	0.0±0.0	26.2±0.0	2.3±0.3	2.4±0.4	1.4±0.2	3.6±1.0

### B. Statistical intercomparison of examined treatment modalities

**Figure 4:**
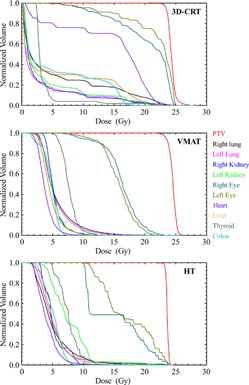
The dose‐volume histograms (DVH) of the PTV and organs at risk (OAR) for the 3D CRT (upper), VMAT (middle), and helical tomotherapy (HT) (lower) radiation modalities.

**Figure 5:**
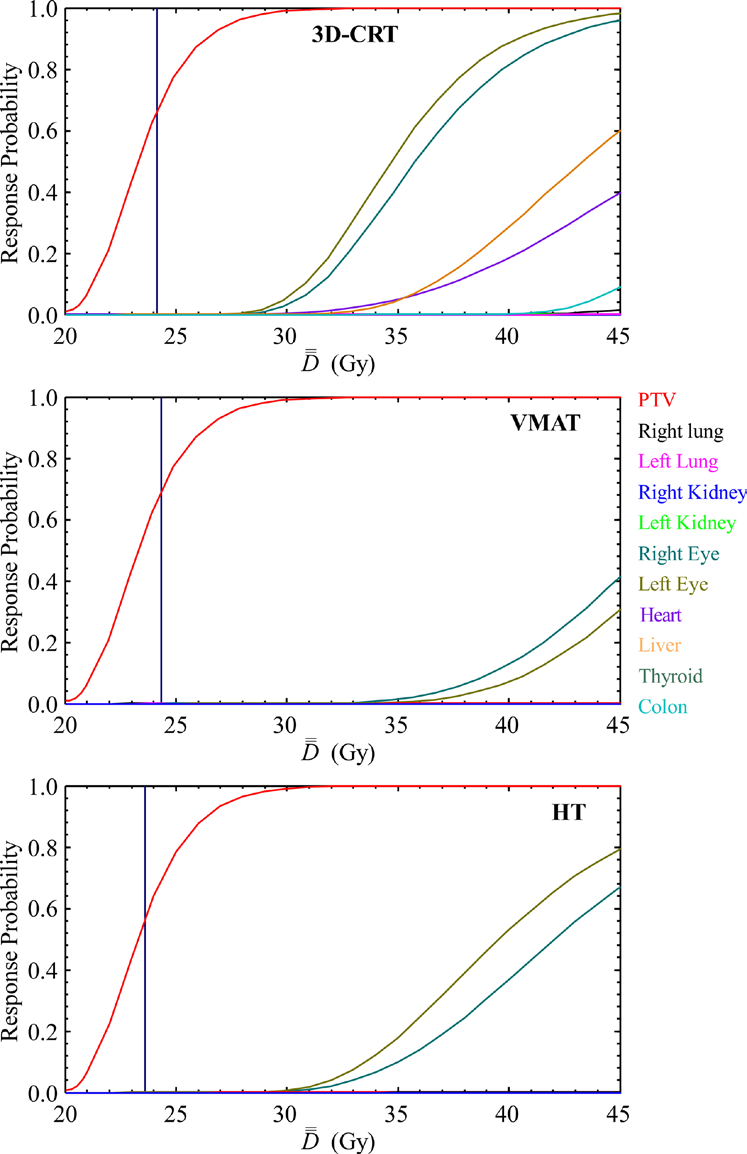
The dose response curves of the PTV and organs at risk (OAR) for the 3D CRT (upper), VMAT (middle), and helical tomotherapy (HT) (lower) radiation modalities. The vertical line indicates the initially prescribed dose level of the respective dose distributions. The biologically effective uniform dose ($\bar{\bar{D}}$) is used in the dose axis in order to make to comparison of the different diagrams compatible using the same scale.

The upper panel of Table 5 summarizes the values of the calculated radiobiological quantities averaged over the 24 patients for each treatment modality and their respective standard deviation. Overall, the average values of $P_{+}$ and $P_{B}$ for the three modalities (approximately 57%–67%) were found to reasonably agree with other published data.^31^, ^32^, ^33^ Using a paired *t*‐test (p < 0.05), it was found that there was a significant difference between the $P_{+}$ values of VMAT and 3D CRT (p = 0.004), as well as between the values of VMAT and those of HT (p < 0.0001). When comparing the 3D CRT and HT modalities, the $P_{+}$ values were significantly higher for the 3D CRT plans (p < 0.0001). The overall data show that HT delivers the lowest average $\bar{\bar{D}}$ (23.6 Gy) compared to 3D CRT and VMAT (24.1 and 24.2 Gy, respectively). The lower target $\bar{\bar{D}}$ is due to the steeper dose fall‐off around the PTV for the HT plans, which is indicative of a more homogeneous target dose closer to the prescribed dose. This lower $\bar{\bar{D}}$ results in an overall $P_{+}$ value that is lower than those of the other two modalities. If the HT prescription was increased by approximately 0.5 Gy, thereby effectively increasing the $\bar{\bar{D}}$ for HT plans, the respective $P_{+}$ value would increase and become more comparable to those of the VMAT plans. Figure 4 shows one representative patient's example DVHs of the PTV and OARs for the 3D CRT, VMAT, and HT radiation modalities. These diagrams indicate a steeper PTV curve for the HT plans and show that, if the HT prescription was increased by approximately 0.5 Gy, the $P_{+}$ value would increase and become more comparable to that of the VMAT plans. Figure 5 displays plots from one patient's results displaying the probability of response (either benefit for PTV or injury for OARs) versus $\bar{\bar{D}}$. Figure 5 also contains vertical lines around 23–24 Gy, which indicate the value for the initial $\bar{\bar{D}}$ calculated and reported for each planning modality.

**Table 5:** Radiobiological values averaged over all 24 patients for each technique and associated standard deviation. The results are presented for the applied dose prescription (upper panel), as well as for the radiobiologically optimized dose prescription (lower panel) of the individual cases. A statistical comparison of the $P_{+}$, $P_{B}$, $P_{I}$, and $\bar{\bar{D}}$ values between the different treatment modalities is also shown

*Parameter/Modality*	*3D CRT*		*VMAT*	*HT*
*Applied Dose Prescription*				
$\bar{\bar{D}}$ (Gy)	24.1±0.1		24.2±0.2	23.6±0.1
$P_{+}$ (%)	64.6±3.4		67.4±2.8	56.6±1.4
$P_{B}\;(\%)$	65.2±1.4		67.4±2.8	56.6±1.4
$P_{I}\;(\%)$	0.6±2.7		0.0±0.0	0.0±0.0
*Intercomparison*	$P_{+}$	$P_{B}$	$P_{I}$	$\bar{\bar{D}}$
3D‐CRT vs. VMAT	0.004	<0.001	0.32	<0.001
3D‐CRT vs. HT	<0.001	<0.001	0.32	<0.001
VMAT vs. HT	<0.001	<0.001	1.00	<0.001
*Radiobiologically Optimized Dose Prescription*				
$\bar{\bar{D}}$ (Gy)	28.4±1.4		33.0±1.7	31.9±1.8
$P_{+}$ (%)	94.1±9.4		99.5±0.8	99.3±0.5
$P_{B}\;(\%)$	95.7±7.1		99.7±0.4	99.6±0.3
$P_{I}\;(\%)$	1.6±2.4		0.2±0.4	0.3±0.3
*Intercomparison*	$P_{+}$	$P_{B}$	$P_{I}$	$\bar{\bar{D}}$
3D‐CRT vs. VMAT	0.01	0.02	0.01	<0.001
3D‐CRT vs. HT	0.01	0.02	0.02	<0.001
VMAT vs. HT	0.22	0.22	0.40	0.005

### C. Graphical illustration of the qualities of examined treatment modalities

**Figure 6:**
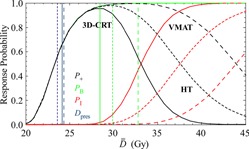
The response curves of the total tumor control probability ($P_{B}$), total normal tissue complication probability ($P_{I}$), and complication‐free tumor control probability ($P_{+}$) for the 3D CRT (solid lines), VMAT (long dashed lines), and helical tomotherapy (HT) (short dashed lines) radiation modalities. The vertical lines on the left indicate the initially prescribed dose level of the respective dose distributions, whereas the vertical lines on the right indicate the prescribed doses that give the optimum radiobiological results for each modality, respectively. The biologically effective uniform dose ($\bar{\bar{D}}$) is used in the dose axis in order to make to comparison of the different diagrams compatible using the same scale.

In Fig. 6, at the point of intersection between the vertical lines and the $P_{+}$ curves, the initially prescribed values of $P_{+}$ and $\bar{\bar{D}}$ are reported for the plans of the three radiation modalities. Figure 6 shows the optimal $\bar{\bar{D}}$ versus $P_{+}$ plots generated by the MATLAB code for the 3D CRT, VMAT, and HT plans for one of the patients. The vertical lines on the left side of the diagram indicate the initial values of $\bar{\bar{D}}$, whereas the vertical lines on the right side indicate the optimal prescribed dose levels. This type of diagram can better illustrate the conformality of the examined treatment modalities. Since the $P_{B}$ curves coincide, differences from the $P_{I}$ curves will indicate which plan is more conformal to the PTV while more effectively sparing the OARs. For example, by selecting a given $\bar{\bar{D}}$ dose level, the $P_{B}$ value will be the same for all the modalities, and the value of $P_{I}$ will point out the which one is the best overall as that with the lowest $P_{I}$ value at that dose level. At low‐dose prescriptions, the differences between the different modalities is shown to be small (due to the negligible $P_{I}$ values for all techniques), but at higher dose prescriptions their differences in quality are able to be determined.

## IV. DISCUSSION

A retrospective analysis of survival of 103 children with primary brain tumors (mean age: 7.6 years) was performed by Araujo and colleagues.^(33)^ In their study, most patients had medulloblastomas or PNET, or low‐grade astrocytomas (18%) and the five‐year survival was found to be 84% for low‐grade astrocytomas and 51% for medulloblastomas and PNET. Respectively, another study using a cohort of 82 patients treated for primary central nervous system (CNS) malignancies (mean age: 6.8 ± 3.39 years) showed that the five‐year overall and event‐free survivals (EFSs) were approximately 68.5% and 35%, respectively.^(34)^ The low‐grade astrocytoma had significantly better outcome than the high‐grade one. Another study included sixty‐six patients with histologically confirmed medulloblastomas (64% pediatric and 36% adults).^(35)^ All the patients underwent postoperative craniospinal irradiation (CSI) delivering a median craniospinal dose of 35.5 Gy with additional boosts to the posterior fossa up to 54.0 Gy. The overall survival (OS) and local and distant progression‐free survival (LPFS and DPFS) were 73%, 62%, and 77% at 60 months. The addition of chemotherapy did not improve survival rates and toxicity was moderate. These are some of the few studies that have been performed recently to provide response data, which can be used for the determination or adjustment of the radiobiological parameters of the models. However, in most of the cases, only limited dosimetric information is reported, especially regarding the three‐dimensional doses delivered to the organs at risk.

In the present study, the relative effectiveness of 3D CRT, VMAT, and HT was examined and the findings indicate that VMAT produce dose distributions that have a higher likelihood to spare the involved OARs while controlling the tumor.

The average data indicated that the total probability of normal tissue complications ($P_{I}$) was low for all the plans, but comparatively, the 3D CRT plans gave the highest $P_{I}$ values. The response values do indicate that the two IMRT techniques, VMAT and HT, are expected to have a lower overall risk of injury for a given patient for the same effective prescribed dose ($\bar{\bar{D}}$). Although the values of BED and ${\text{EQD}}_{2\text{Gy}}$ differ from the corresponding absolute values of $\bar{\bar{D}}$, they show very similar relative differences between the different treatment modalities. The differences are mainly observed in the tissues showing negligible complication probabilities. In these cases, there is a wide range of uniform doses producing the same probability of response. Furthermore, BED is related with the physical doses in the different parts of a tissue with a linear relationship, whereas has a sigmoid relationship with physical dose since dose‐response curves are usually sigmoid. Due to the fact that the values of $\bar{\bar{D}}$ are directly associated with the response probabilities of the corresponding tissues, the similar relative behavior of $\bar{\bar{D}}$ with that of BED verifies and supports the conclusions obtained by the relative radiobiological comparisons.

Analysis of Fig. 6 also indicates a wider “therapeutically beneficial range” for VMAT and HT. The therapeutically beneficial range is indicated by the width of the $P_{+}$ curve. The wider the bell shape of this curve, the larger the range of the prescription dose that can be delivered without an additional increase in the probability of injury. This greater window will allow the achievement of a higher benefit without compromising the selection of the prescribed dose in order to avoid additional complications. By examining the average $P_{+}$ values calculated using the actual plan DVHs, the study found that the VMAT plans delivered the highest probability of benefit while maintaining the lowest probability of injury, as compared with the other two techniques. In addition to calculating the radiobiological parameters at the initially prescribed dose (as planned from the TPS), the MATLAB code calculates the optimal $P_{+}$ and the corresponding $P_{B}$, $P_{I}$, and $\bar{\bar{D}}$.

In the radiobiological diagrams, the maximum $P_{+}$ is identified and the corresponding value of $\bar{\bar{D}}$ on the x‐axis can be determined. This data can be very useful in determining whether or not the prescribed dose level is optimal for the patient based on the expected radiation effects. If the optimal $\bar{\bar{D}}$ is higher or lower than the initial $\bar{\bar{D}}$ of a given plan, it shows that the prescription could be increased or decreased in order to maximize the $P_{+}$ and, therefore, allow the patient to receive the maximum benefits of the radiation therapy. The lower panel of Table 5 summarizes the average optimal results of the radiobiological quantities for each treatment modality over all the patients and respective standard deviation. At this point it should be clarified that the effectiveness of a given dose distribution is evaluated by the comparison of its advantages in terms of tumor control against its disadvantages regarding normal tissue complications.^6^, ^11^ The original definition of $P_{+}$ does not incorporate weights between the targets and OARs, nor between the different OARs. In clinical practice, there are not different weighting factors that are applied, but there are risk thresholds (e.g., 5%–10%) for every organ at risk, which should not be exceeded. So, in order to classify the different treatment plans, one can, instead of using the $P_{+}$ index, select in the diagrams of 5, 6 the dose level that satisfies the demands imposed by the normal tissues risk thresholds and associate them with the expected tumor control rate at this dose level. The later way of classifying the plans is more appropriate when additional clinical endpoints need to be considered, such as neurocognitive toxicity, the need of growth hormone replacement or the risk for secondary malignancies.

In this work, the risks for acute only radiation effects to the organs at risk were used for the estimation of the overall NTCP, which are the ones used during treatment planning. However, the risk for secondary malignancies should also be considered in the future given that the uncertainties characterizing their parameters will be reduced at least to the levels of the other endpoints. The need for estimating the risk for secondary malignancies is imposed by the young age of the patients and the large low‐dose regions characterizing the HT and VMAT modalities (see Fig. 3). Spreading of low dose may be a major limitation which needs to be addressed, and alternative planning methods (such as partial arcs) may need to be pursued.

Regarding the optimal prescription, the radiobiological analysis indicates equivalents between the three radiation modalities even though the dosimetric data indicate superiority of VMAT and helical tomography over 3D CRT. However, knowing the VMAT and HT have a higher a number of degrees of freedom in delivering a treatment, a difference could potentially be seen if their treatment plans had initially been optimized based on radiobiological measures. The optimal data indicate the possibility for a very high probability of controlling the tumor without causing severe injury to the normal tissues, $P_{+}$, for all the three modalities. The two IMRT techniques, VMAT and HT, show similar $P_{+}$ values of over 99% with very low probabilities of injury (less than 0.3%). Using a paired *t*‐test (p < 0.05), there was a significant increase in $P_{+}$ for both IMRT methods when comparing them against the 3D CRT technique (p = 0.01 for VMAT vs. 3D CRT and p = 0.01 for HT vs. 3D CRT). The 3D CRT plans have a lower maximum $P_{+}$ value due to the increased probability of injury associated with it. There was no significant difference between the HT and VMAT methods based on the comparison of their maximum $P_{+}$ values (p = 0.22) and the corresponding $P_{B}$ and $P_{I}$ values (p = 0.22 and 0.40, respectively). This trend is also evident in 5, 6. The therapeutically beneficial range of the $P_{+}$ curve for the 3D CRT plans is narrower as compared to the other two modalities. The optimal doses found for all three modalities indicate that a higher prescribed dose may be used to increase the probability of tumor control while maintaining a low risk of injury. This study indicates that using either IMRT modality (VMAT or HT) for pediatric CSI will yield a higher probability for benefit for these pediatric patients, as compared with the 3D CRT. It should be stated here that the values reported here do not serve for absolute response comparisons (especially regarding tumor response), but, for the purposes of this study, they serve to show the relative impact of using the appropriate technique regarding the expected overall treatment outcome. The determination of dose‐response parameters for many of the organs is still very uncertain in pediatric patients. However, the values used, do not affect the relative comparison between the three treatment techniques that were examined.

Currently this method of calculating the “optimal” dose prescription is not used in the clinic, as physicians prescribe a preset dose level to the target based on known values from literature. In the future, however, radiobiogically driven methods of dose prescription may become more prevalent, and the secondary conclusion of this study may prove to be an important finding.

All the radiobiological parameters (e.g., $D_{50}$, γ, s, and α/β) that are used to calculate the tissue responses are characterized by large uncertainties (stemming from, for example, inherent deficiencies of the model, inter‐ and intrapatient radiosensitivity variations, discrepancies between plan and delivery). Consequently, it is more proper to treat the findings of these studies and comparisons, as well as of the present one, as relative trends between the different radiation modalities that are compared rather than absolute expected values. In this sense, their relative optimization could be a useful feature in treatment planning and treatment technique comparisons.

The differences usually observed between different models stem from their inherent structural differences, but also from the variation in the values of their radiobiological parameters since it is very rare to find parameter values for different models which have been derived from the same patient material.^(18,36‐37^) In the present study, the choice of model or the uncertainties characterizing the relevant radiobiological parameters may play a significant role in the estimation of the NTCP results. Especially, since there is no consensus in the literature on the validity of different NTCP models, it is apparent that these models should be used with great caution. Consequently, the presented results are valid only for the radiobiological models and parameter values used in this particular work for the calculation of the TCP and NTCP values of the three radiation modalities.

Radiobiological measures are closer associated with the clinical outcome even though their clinical implementation is limited due to a number of factors that are gradually eliminated. One of these factors is the large uncertainties that usually characterize the parameters describing the dose‐response relations of the different tumors and normal tissues. These parameters are extracted from patient materials where the dose delivered to each patient and the follow‐up records are available.^(22,24^) The values of the parameters are influenced by the variation of the treatment methodologies among different institutions and the limitations in the clinical information used, such as imaging at cellular level, accurate determination of the dose delivered to the patient, and radiosensitivity of the individual patient. The radiobiological parameter values applied to a certain patient material should be compatible with the clinical characteristics of the patients at hand. By using such a set of parameters, the order of the relative difference in the effectiveness between the dose distributions of different radiation modalities could be estimated in terms of treatment outcome. However, in order to estimate the absolute response rates with a clinically acceptable accuracy, the derivation of the radiobiological parameters from own clinical data is inevitable. In this way, they can be validated for their compatibility with the examined patient population.

## V. CONCLUSIONS

A radiobiological treatment plan analysis is considered to be more complete because it takes into account more biological mechanisms that are associated with the effects of radiation to the different tissues. In this way, it shows a greater potential to estimate the treatment outcome compared to the conventional dosimetric treatment plan analysis. The dosimetric information combined with the radiobiological analysis (based on the $\bar{\bar{D}}$ and $P_{+}$ quantities) may lead to better evaluation of the quality of a treatment plan. This study aimed at assessing the relative effectiveness of the 3D CRT, VMAT, and helical tomotherapy treatment modalities regarding the treatment of pediatric CSI patients. As a result of analyzing the planned dose distributions of these three modalities for 24 patients, the VMAT plans were found to have the highest complication‐free tumor control probability as compared to the other two modalities regarding the current clinical prescription, even though the differences render the three modalities almost equivalent (especially for the optimal dose prescription). Another finding of this study concerns the potential for dose escalation, which may improve the effectiveness of the three examined modalities, especially the VMAT. While currently this method of dose prescription is not used clinically, it may become more relevant as radiobiological treatment planning becomes more widely implemented in the clinical routine. Although precise, conclusive dose‐response data may be limited for pediatric cases, this study presents a relative evaluation concluding that the techniques that are capable of delivering the most effective treatments to children treated with CSI are the two IMRT modalities of VMAT and helical tomotherapy.
